# Differences in outcome according to *Clostridium difficile* testing method: a prospective multicentre diagnostic validation study of *C difficile* infection

**DOI:** 10.1016/S1473-3099(13)70200-7

**Published:** 2013-11

**Authors:** Timothy D Planche, Kerrie A Davies, Pietro G Coen, John M Finney, Irene M Monahan, Kirsti A Morris, Lily O'Connor, Sarah J Oakley, Cassie F Pope, Mike W Wren, Nandini P Shetty, Derrick W Crook, Mark H Wilcox

**Affiliations:** a Centre for Infection and Immunity, Division of Clinical Medicine, St George's, University of London, London, UK; b Department of Medical Microbiology, St George's Healthcare NHS Trust, London, UK; c Microbiology Department, Leeds Teaching Hospitals NHS Trust and University of Leeds, Leeds, UK; d Clinical Microbiology and NIHR OxBRC Infection Theme, Oxford University Hospitals NHS Trust, Oxford, UK; e Infection Control, University College London Hospitals NHS Foundation Trust, London, UK; f Department of Clinical Microbiology and Virology, Health Protection Agency and University College London Hospitals NHS Foundation Trust, London, UK

## Abstract

**Background:**

Diagnosis of *Clostridium difficile* infection is controversial because of many laboratory methods, compounded by two reference methods. Cytotoxigenic culture detects toxigenic *C difficile* and gives a positive result more frequently (eg, because of colonisation, which means that individuals can have the bacterium but no free toxin) than does the cytotoxin assay, which detects preformed toxin in faeces. We aimed to validate the reference methods according to clinical outcomes and to derive an optimum laboratory diagnostic algorithm for *C difficile* infection.

**Methods:**

In this prospective, multicentre study, we did cytotoxigenic culture and cytotoxin assays on 12 420 faecal samples in four UK laboratories. We also performed tests that represent the three main targets for *C difficile* detection: bacterium (glutamate dehydrogenase), toxins, or toxin genes. We used routine blood test results, length of hospital stay, and 30-day mortality to clinically validate the reference methods. Data were categorised by reference method result: group 1, cytotoxin assay positive; group 2, cytotoxigenic culture positive and cytotoxin assay negative; and group 3, both reference methods negative.

**Findings:**

Clinical and reference assay data were available for 6522 inpatient episodes. On univariate analysis, mortality was significantly higher in group 1 than in group 2 (72/435 [16·6%] *vs* 20/207 [9·7%], p=0·044) and in group 3 (503/5880 [8·6%], p<0·001), but not in group 2 compared with group 3 (p=0·4). A multivariate analysis accounting for potential confounders confirmed the mortality differences between groups 1 and 3 (OR 1·61, 95% CI 1·12–2·31). Multistage algorithms performed better than did standalone assays.

**Interpretation:**

We noted no increase in mortality when toxigenic *C difficile* alone was present. Toxin (cytotoxin assay) positivity correlated with clinical outcome, and so this reference method best defines true cases of *C difficile* infection. A new diagnostic category of potential *C difficile* excretor (cytotoxigenic culture positive but cytotoxin assay negative) could be used to characterise patients with diarrhoea that is probably not due to *C difficile* infection, but who can cause cross-infection.

**Funding:**

Department of Health and Health Protection Agency, UK.

## Introduction

*Clostridium difficile* infection is usually health-care associated, is related to antibiotic use, and usually manifests as diarrhoea. The infection causes an estimated 3000 deaths every year in the UK and 15 000–20 000 deaths in the USA,^1^, ^2^ with associated case-fatality rates of 6–17%.^3^, ^4^, ^5^, ^6^ It is associated with the overgrowth of *C difficile* and the production of toxins A or B, or both, which cause a range of effects, including gut mucosal damage, colitis, and pseudomembranous colitis.

Since the features of health-care-associated diarrhoea cannot reliably distinguish *C difficile* from other causes, laboratory confirmation is essential. However, optimum laboratory diagnosis of *C difficile* infection remains controversial.^7^, ^8^, ^9^, ^10^, ^11^ Two reference methods exist: the cell cytotoxicity assay, which detects neutralisable toxins; and cytotoxigenic culture, which establishes whether cultured *C difficile* isolates can produce toxin in vitro. Crucially, the reference methods detect different targets, and so cytotoxigenic culture might be regarded as having inadequate specificity,^9^, ^12^ or toxin detection as having insufficient sensitivity.^9^, ^13^ However, the significance of a positive result with either of these methods is unclear, and in turn hinders the clinical interpretation and validation of diagnostic methods for *C difficile* infection.^12^ The availability of many commercial tests with different *C difficile* targets is both indicative of, and contributes to, uncertainty. The widely available assays are *C difficile* toxin A and B enzyme immunoassays that detect free toxin in faeces,^14^, ^15^ glutamate dehydrogenase tests that detect a common antigen produced by *C difficile*, and nucleic acid amplification tests that detect toxin genes but not free toxin.

The performances of *C difficile* toxin enzyme immunoassays are inadequate, with positive predictive values less than 50% in settings of low disease prevalence.^14^, ^15^, ^16^ Glutamate dehydrogenase assays have been proposed as the first step in two-stage algorithms for diagnosis of *C difficile* infection,^17^, ^18^, ^19^, ^20^ but their low specificity makes them unsuitable as standalone tests. Several toxin gene nucleic acid amplification tests are available, but are also not ideal as standalone tests; they are quite expensive (about five to ten times more expensive than enzyme immunoassay) and the lower 95% CI for sensitivity is roughly 80–85% and that for specificity is about 93%.^12^ Since available diagnostic assays seem to be inadequate as standalone tests, several two-stage or three-stage algorithms have been proposed.^17^, ^18^, ^19^, ^20^, ^21^ However, most studies comparing *C difficile* tests and algorithms have neither assessed each potential target nor used both reference methods, and have relied on low sample numbers (usually from a single centre), leading to unacceptably wide confidence intervals.^17^, ^18^, ^19^, ^21^ A recent survey of UK diagnostic laboratories drew attention to the absence of consensus about the optimum algorithm when it showed that more than 25 different algorithms for *C difficile* infection are now in use.^22^

Imprecise diagnosis has implications for infection control practice, patient management, and performance management of institutions.^7^, ^12^ The situation is exacerbated because health-care-associated diarrhoea often results from causes other than *C difficile* infection, including other infections (eg, norovirus), antibiotics, laxatives, or surgery. To address these shortcomings, we undertook a large observational diagnostic study in four routine diagnostic laboratories, in which we used routinely submitted diarrhoeal faecal samples. We aimed to clinically interpret the reference methods for *C difficile* infection, and to assess *C difficile* test performance with sufficient accuracy to compare single assays and establish the best possible algorithm for the laboratory diagnosis of *C difficile* infection.

## Methods

### Study design

We did this study in four UK hospital diagnostic laboratories serving Leeds Teaching Hospitals NHS Trust, St George's Healthcare NHS Trust, Oxford University Hospitals NHS Trust, and University College London Hospitals NHS Foundation Trust, and their respective communities. The four study hospitals are major teaching hospitals, each covering the following specialties: general medicine and surgery, elderly patients, children, renal, transplant, and oncology. The average testing rate in these hospitals was 142·7 tests per 10 000 patient bed days (range 105–156 per 10 000).

We did all assays on all specimens during a training phase (October, 2010–April, 2011). We established the optimum algorithm for each reference method and carried it forward to the testing phase (May–September, 2011). We tested samples in the training and testing phases with both reference methods. The study was approved by the National Research Ethics Service (reference number 10/H0715/34).

### Samples and procedures

Faecal samples from both hospital and community patients submitted for routine testing for *C difficile* were eligible for inclusion. We followed the routine protocol for sample submission and *C difficile* testing in the UK. We tested all unformed faecal samples (Bristol stool chart types 5–7, not clearly attributable to an underlying disease or treatment) from all hospital patients (aged ≥2 years) and from individuals in the community (aged ≥65 years), irrespective of *C difficile* or other testing requests. Samples were stored at 2–5°C and analysed within 5 days. We did all assays at the site of submission on unblinded samples.

Methods were standardised across all sites with a laboratory manual and standard operating procedures, and every evaluator was fully trained in the two reference methods before the study began. Cell cytotoxin assay and cytotoxigenic culture were done with standard laboratory methods. Cell cytotoxin assay was identified by detection of cytopathic effect on Vero cells, cultured for 48 h, which is abolished by antitoxin. Cytotoxigenic culture was done after alcohol shock by anaerobic culture on Brazier's agar for 48 h. *C difficile* was then identified and broth cultured for a further 48 h and tested for cell cytotoxin (appendix).

For quality assurance, every month one participating laboratory, on rotation, sent six blinded samples to the other three sites. All assays were undertaken on the samples and results compared for inter-laboratory variation.

We assessed tests that represent the three main targets for *C difficile* detection: bacterium (glutamate dehydrogenase), toxins, or toxin genes. Training was provided by assay manufacturers and all tests were done according to their instructions. The enzyme immunoassays were automated and done on DS2 instruments (Magellan Biosciences, North Billerica, MA, USA). We assessed the following assays: Meridian Premier toxins A&B enzyme immunoassay (toxin enzyme immunoassay 1; Meridian Bioscience, Cincinnati, OH, USA); Techlab *C difficile* Tox A/B II toxin enzyme immunoassay (toxin enzyme immunoassay 2; Techlab, Blacksburg, VA, USA); and the Techlab *C diff* Chek-60 glutamate dehydrogenase enzyme immunoassay (Techlab). The nucleic acid amplification test assay was GeneXpert (Cepheid, Sunnyvale, CA, USA).

We gathered routinely available patient data as follows: blood test results obtained within 3 days of faecal samples (white cell count, and serum creatinine and albumin concentrations), dates of admission and discharge, age, sex, and 30-day all-cause mortality. We obtained baseline serum creatinine concentrations from tests done more than 6 months before study entry.

We recorded additional data, including computerised tomographic evidence of colitis, or if *C difficile* was mentioned on a death certificate, for patients with positive cytotoxigenic culture, cell cytotoxin assay, or nucleic acid amplification tests. UK death certificates have two sections: part 1, cause of death; and part 2, contributing to but not causing death. In all cases that were cytotoxigenic culture positive and cell cytotoxin assay negative, we ascertained the diagnosis of the attending team; whether *C difficile* treatment was given; and the duration, frequency, and consistency of diarrhoea.

Data were collected, anonymised, and then sent to a central coordinator. Data queries were resolved and the database was locked on Jan 25, 2012.

### Management of *C difficile* infection

Treatment of *C difficile* infection followed standard guidelines and remained unchanged during the study. Oral metronidazole was given to non-severe cases and oral vancomycin to severe cases, with the exception that all patients in one centre (Oxford) received oral vancomycin.

Routine laboratory testing methods and reporting for *C difficile* continued during the study period. Relevant additional positive study results were reported to clinicians. Since a cytotoxigenic culture result can take up to 5 days to obtain, this test often had little or no effect on immediate case management.

### Analyses

A predefined analysis plan categorised patients into three groups: group 1, cell cytotoxin assay positive; group 2, cytotoxigenic culture positive and cell cytotoxin assay negative; and group 3, both cell cytotoxin assay and cytotoxigenic culture negative. Potential risk factors included were hospital site, age, sex, time in hospital before the stool sample was taken, albumin concentration lower than 20 g/L, white cell count greater than 15 × 10^9^ per L, and a greater than 50% rise in serum creatinine concentration. Patients with any one or more of the following were predefined to represent severe disease: white cell count greater than 15 × 10^9^ per L, a greater than 50% rise in serum creatinine concentration, CT or other evidence of colitis, and an albumin concentration less than 20 g per L.

Post-diagnosis survival was defined as alive at discharge or 30 days after diagnosis (whichever was earlier). Since several faecal samples were often obtained for every patient, one sample was used per episode of diarrhoea, which was defined as a diarrhoeal sample received more than 28 days after a previous sample. For the analysis, we used the first sample positive for *C difficile* by either reference method, or the first negative sample if no sample was positive. We regarded the inpatient time before diarrhoea as a potential risk factor for *C difficile* infection.

### Statistical analysis

The sample size calculations were made with the assumption of an inpatient positivity rate of 4·5%, with 16% of these patients cytotoxigenic culture positive and cell cytotoxin assay negative. We calculated that 18 000–20 000 samples were needed to detect a 15% difference in mortality between cell cytotoxin assay positive cases and those that were cytotoxigenic culture positive but cell cytotoxin assay negative. Preplanned interim analysis showed a higher positivity rate (7·5%, of which roughly 40% were cytotoxigenic culture positive and cell cytotoxin assay negative), which led to a reduction in the sample size needed.

For analysis, we used Stata 12·0. We investigated univariate trends with *t* tests and one-way ANOVA, and Fisher's exact test for categorical data. For multivariate analysis, we used unconditional logistic regression for mortality and parametric survivorship analysis for lengths of hospital stay. For survivorship analysis, we used the exponential parametric model and censored observations at death or discharge (whichever occurred first).^23^ We started with the full model and removed non-significant variables in a stepwise manner. To assess model–data fit, we used the Hosmer-Lemeshow test.^24^ To investigate excess mortality associated with some diagnostic groups we used bootstrap sampling, and confidence intervals were corrected for bias.^25^

We calculated sensitivity, specificity, positive predictive value, and negative predictive value for single assays and combinations of assays (algorithms) in the training phase. We analysed all the samples included, whether a single patient had several samples or not. Interclass correlation coefficients and repeat analyses on de-duplicated datasets were done (appendix). Adequate assay performance was defined as greater than 90% sensitivity and greater than 99·5% specificity. We established the optimum algorithm for each reference method and used this method in the testing phase. We used second stage assays only on selected samples (those positive in the first assay), and so could not calculate sensitivity and specificity data from this dataset. We compared the two reference methods for agreement (kappa). Assays and algorithms were compared by analysis of area under receiver operator characteristics curve (AUROC). For this calculation we used Fawcett's exact method^26^ and the bootstrap was used for calculation of 95% CIs.^25^ We plotted time series to study positivity rates for each assay overall and for each participating centre.

### Role of the funding source

The sponsor of the study had no role in study design, data collection, data analysis, data interpretation, or writing of the report. The corresponding author had full access to all the data in the study and had final responsibility for the decision to submit for publication.

## Results

12 420 samples were tested from 10 691 diarrhoeal episodes in 10 186 patients between Oct 15, 2010, and Sept 29, 2011. The training and testing datasets comprised 6753 and 5667 samples, respectively. 5197 (42%) samples were from Leeds, 3852 (31%) from Oxford, 1778 (14%) from University College Hospital, and 1593 (13%) from St George's Hospital.

**Figure 1:**
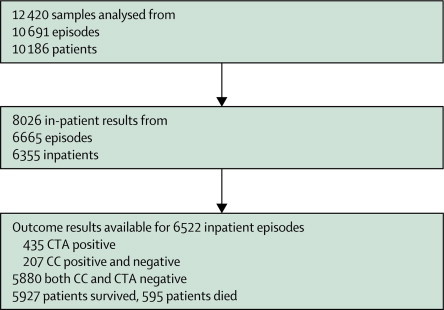
Patient and sample selection CC=cytotoxigenic culture. CTA=cytotoxin assay.

We obtained 8026 results from 6665 episodes in 6355 inpatients. Outcome data were available for 6522 inpatient episodes with reference assay results available (6283 patients; figure 1). Table 1 shows baseline characteristics. 926 first samples (either inpatient or outpatient) were positive by either reference method, 620 were positive by both, 306 by cytotoxigenic culture only, and 56 by cell cytotoxin assay only (κ 0·739, 95% CI 0·721–0·578). Reference method test results were unavailable for two of 6524 patient episodes (because of unresolved results). Therefore, 6522 inpatient episodes with outcome data were available for analysis (table 2).

**Table 1:** Baseline characteristics of inpatients at the four study sites

	**All patients**	**Site 1**	**Site 2**	**Site 3**	**Site 4**
Episodes	6665	2635	1368	1092	1570
Inpatients	6355	2560	1280	1025	1490
Female patients	3582/6661	1462/2635	666/1364	592/1092	862/1570
Age (years)	64 (21)	63 (22)	59 (21)	67 (20)	68 (20)
White cell count ×10^9^/L	10·0 (10·3)	10·3 (14·1)	8·8 (6·2)	10·0 (5·5)	10·7 (9·1)
White cell count >15×10^9^/L	940/6204	383/2322	147/1328	143/1056	267/1498
Serum creatinine concentration, μmol/L	106·5 (98·7)	110·9 (91·6)	86·4 (74·7)	106·0 (97·1)	118·0 (123·9)
Rise in serum creatinine concentration (%)	33·9% (85·8)	18·8% (55·9)	6·89% (52·6)	40·9% (93·1)	68·0% (112·6)
Albumin concentration, g/L	32·8 (7·7)	34·7 (6·8)	32·5 (7·6)	26·3 (7·4)	35·1 (6·4)
Albumin <20 g/L	270/5500	21/1908	60/1292	182/968	7/1332
Deaths	595/6524	283/2634	68/1232	125/1088	119/1570
CT positive	615/6663	275/2634	84/1367	99/1092	157/1570
CTA positive	446/6663	236/2634	51/1367	57/1092	102/1570

Data are n, n/N, or mean (SD). The denominators vary in the table because of occasional missing values. Site 1=Leeds Teaching Hospitals NHS Trust. Site 2=University College London Hospitals NHS Foundation Trust. Site 3=St George's Healthcare NHS Trust. Site 4=Oxford University Hospitals NHS Trust. CTA=cytotoxin assay.

**Table 2:** Clinical characteristics of first episodes of inpatients with available clinical outcome results

	**Group 1 (CTA positive)**	**Group 2 (CC positive, CTA negative)**	**Group 3 (all negative)**	**Group 1 *vs* group 2 p values**	**Group 1 *vs* group 3 p values**	**Group 2 *vs* group 3 p values**
n	435	207	5880	..	..	..
Female (%)	243/435 (56%)	118/207 (57%)	3154/5876* (54%)	..	..	..
Mean age, (years; SD)	69 (20)	66 (21)	64 (21)	..	..	..
Mean white cell count (×10^9^/L; SD)	12·4 (8·9)	10·1 (5·8)	9·9 (10·7)	0·0004	<0·0001	0·6970
Mean rise in creatinine (%; SD)	37% (63)	52% (147)	33% (85)	0·1238	0·2270	0·0028
>100% rise in creatinine (%)	40/316 (13%)	21/169 (12%)	447/4729 (9%)	..	..	..
Mean albumin (g/L; SD)	31 (7)	32 (8)	34 (8)	0·5226	<0·0001	0·0017
Albumin <20 g/L (%)	13/344 (4%)	11/166 (7%)	241/4855 (5%)	..	..	..
Died (%)	72/435 (16·6%)	20/207 (9·7%)	503/5880 (8·6%)	0·022	<0·0001	0·530
Mean length of stay before sample (days; SD)	18·0 (29)	14·1 (24)	10·7 (21)	0·1584	<0·0001	0·0157
Mean length of stay after sample (days; SD)	19·4 (25)	18·6 (27)	14·2 (22)	0·9498	<0·0001	0·0022
Death rate per 1000 inpatient days	9·03	5·33	6·26	0·0195	0·0033	0·4224

CTA=cytotoxin assay. CC=cytotoxigenic culture.

* Sex was not recorded for four patients in this group.

595 deaths were recorded within 30 days. The 30-day case all-cause mortality rate was significantly higher in group 1 than in groups 2 or 3 (table 2); however, the all-cause mortality rate did not differ significantly between groups 2 and 3. These data give a rough excess all-cause mortality rate of 8% (95% CI 4–11) in patients with a positive cell cytotoxin assay. The all-cause mortality rate was not significantly higher in group 2 than in group 1. Expressed as a death rate per 1000 inpatient days, patients in group 1 had significantly higher mortality than did those in groups 2 or 3. The death rate did not differ significantly between groups 2 and 3 (table 2).

We did a similar analysis with a nucleic acid amplification test as a reference method as a surrogate of cytotoxigenic culture. Faecal specimens that were positive by nucleic acid amplification testing but negative by cell cytotoxin assay were no better predictors of a fatal outcome or prolonged length of stay than were cases that were cytotoxigenic culture positive and cell cytotoxin assay negative (table 3).

**Table 3:** Clinical characteristics of first episodes of inpatients with available clinical outcome results with use of the result of the CTA and NAAT tests to define diagnostic categories

	**CTA positive**	**NAAT positive/CTA negative**	**CTA and NAAT negative**	**CTA positive *vs* NAAT positive/CTA negative p value**	**CTA positive *vs* CTA and NAAT negative p value**	**NAAT positive/CTA negative *vs* CTA and NAAT negative p value**
Number	435	311	3943	..	..	..
Female (%)	243/435 (56%)	174/311 (56%)	2117/3941* (54%)	..	..	..
Mean age (years; SD)	69 (20)	64 (22)	64 (21)	..	..	..
Mean white cell count (×10^9^/L; SD)	12·4 (8·9)	9·9 (6·6)	10·0 (12·0)	<0·0001	<0·0001	0·8633
Mean rise in creatinine (%; SD)	37% (63)	49% (132)	34% (81)	0·0222	0·3018	0·0085
>100% rise in creatinine (%)	40/316 (13%)	30/245 (12%)	321/3163 (9%)	..	..	..
Mean albumin (g/L; SD)	31 (7)	33 (8)	33 (8)	0·0328	<0·0001	0·0456
Albumin <20 g/L (%)	13/344 (4%)	15/258 (6%)	166/3223 (5%)	..	..	..
Died (%)	72/435 (16·6%)	30/311 (9·7%)	349/3943 (8·9%)	0·004	<0·0001	0·606
Mean length of stay before sample (days; SD)	17·9 (29)	13·6 (23)	11·2 (22)	0·0311	<0·0001	0·0978
Mean length of stay after sample (days; SD)	19·4 (25)	16·5 (24)	15·1 (24)	0·1869	0·0010	0·2771
Death rate per 1000 inpatient days	9·03	6·04	6·05	0·0317	0·0018	0·8436

CTA=cytotoxin assay. CC=cytotoxigenic culture. NAAT=nucleic acid amplification test.

* Sex was not recorded for two patients in this group.

The following predefined criteria were used in a multivariate logistic regression model with 30-day mortality as the dependent factor: diagnostic category (group 1, 2, or 3), age older than 65 years, white cell count higher than 15 × 10^9^ per L, albumin less than 20 g per L, and the hospital where the sample was taken. Compared with group 3, group 1 (cell cytotoxin assay-positive samples) remained significantly associated with mortality (table 4). Bootstrap analysis applied to this multivariate model showed significant excess mortality between diagnostic groups 1 and 3 (2·95%, 95% CI 0·49–6·07%); there was a 35·8% (95% CI 5·83–52·9) excess of deaths in positive cases—ie, those attributable to *C difficile* infection. All other group comparisons (group 1 *vs* group 2, and group 2 *vs* group 3) were not significant (data not shown).

**Table 4:** Multivariate logistic regression analysis with 30-day mortality as the dependent factor

	**OR (95% CI)**	**p value**
Group 1 *vs* group 3	1·61 (1·12–2·31)	0·0101
Age >65 years	2·52 (1·98–3·21)	<0·0001
Site 2 *vs* site 1	0·48 (0·35–0·67)	<0·0001
Site 4 *vs* site 1	0·54 (0·41–0·72)	<0·0001
WCC >15 × 10^9^/L	1·94 (1·52–2·47)	<0·0001
>50% rise in serum creatinine	2·25 (1·69–2·99)	<0·0001
Serum albumin <20 g/L	2·72 (1·90–3·91)	<0·0001

OR=odds ratio. WCC=white cell count.

Patients in groups 1 and 2 had a longer inpatient stay than did those in group 3 before a stool sample was sent to the laboratory (table 2). The lengths of stay before and after a *C difficile* test (because of diarrhoea) were significantly correlated (correlation coefficient=0·3353, 95% CI 0·2810–0·3897). In univariate analysis (table 2), group 3 patients had a significantly shorter stay in hospital than did those in both groups 1 and 2. The diagnostic group or the result of the *C difficile* diagnostic tests did not predict the length of stay in multivariate logistic regression, independently of other predictors (data not shown).

Additional data were gathered for 797 samples positive for *C difficile* in 710 patients. Of these samples, 446 were positive by cell cytotoxin assay, and of the remaining 351 discordant samples, 176 were positive by both nucleic acid amplification test and cytotoxigenic culture, 34 were positive by cell cytotoxin assay alone, and 141 were positive only by nucleic acid amplification testing. We recorded no difference in the proportion of patients with signs of (predefined) severe disease in group 1 and group 2 (128/247 *vs* 56/130, p=0·13). We also noted no difference in disease severity in group 1 patients compared with those who were cell cytotoxin assay negative but nucleic acid amplification test positive (128/247 *vs* 85/187, p=0·21).

Death certificate data were available for 61 of 92 of the fatal cases that were positive by cell cytotoxin assay and 72 of 102 of those positive by cytotoxigenic culture (or nucleic acid amplification test). *C difficile* was mentioned in part 1 of more death certificates in group 1 than in group 2 patients (nine of 39 *vs* none of 22, p=0·02), and in more parts 1 or 2 of group 1 than group 2 patients (19/39 *vs* three of 22, p=0·01). The differences remained when the analysis was repeated with a comparison of cell cytotoxin assay and nucleic acid amplification test results (data not shown).

At least some additional clinical data were available in 143 of 206 inpatients with discordant reference method results. Of these patients, 75 who were cytotoxigenic culture positive but cell cytotoxin assay negative received no treatment for *C difficile* infection, 37 received metronidazole, 23 vancomycin, and two both drugs. Of the four of 75 cases that were cytotoxigenic culture positive and cell cytotoxin assay negative who died and did not receive treatment for *C difficile* infection, none had a diagnosis of this infection on their death certificate. Only a few (17/138; 12%) of the patients with a discordant reference method result had a recorded clinical diagnosis of *C difficile* infection. Furthermore, 64 of 143 (45%) patients with a discordant reference method result did not have diarrhoea recorded on their stool chart; for the remainder of patients, the median duration of diarrhoea was 2 days (IQR 1·25–5·5).

12 366 samples had results for cell cytotoxin assay and 12 402 for cytotoxigenic culture. Of these samples, 1037 patients had two samples, 199 had three samples, 66 had four samples, and 21 patients had five or more samples. The Xpert assay produced repeat invalid results in 26 samples, which were removed from further analysis. No other assay produced invalid or indeterminate results. The glutamate dehydrogenase and toxin enzyme immunoassay 2 tests were not done on 37 and 33 samples, respectively, because of insufficient faeces. The toxin enzyme immunoassay 1 and Xpert assays were not used first line in the testing phase, and so represent a smaller and partially selected dataset (appendix). Although we noted a strong correlation between repeat samples, this correlation became insignificant when the analysis was repeated on a duplicated dataset and showed no important differences between these estimates of diagnostic performance and those for the entire real-life dataset (appendix).

In the training dataset, no individual assay reached both an adequate level of sensitivity and specificity compared with either reference method (appendix). The performances of the two toxin enzyme immunoassays differed substantially, especially in terms of sensitivity (appendix); median AUROCs for toxin enzyme immunoassay 2 (0·949 [SE 0·0026] and 0·817 [0·0046]) were higher than for toxin enzyme immunoassay 1 (0·906 [0·0035] and 0·791 [0·0050]) compared with cell cytotoxin assay and cytotoxigenic culture (both p<0·0001).

**Figure 2:**
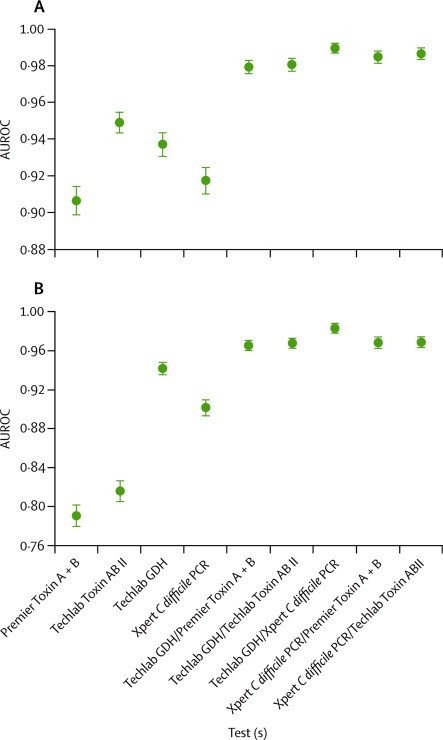
AUROCs (median and 95% CI) for each individual assay and algorithms (A) Comparison with the cell cytotoxicity reference method. (B) Comparison with the cytotoxigenic culture reference method. Comparisons were done in the training phase. Error bars represent 95% CIs. AUROC= area under the receiver operator characteristic curve. GDH=glutamate dehydrogenase.

An interim analysis of the training set showed that no algorithm or assay was best according to both reference methods, although algorithms performed better than individual assays (figure 2). The optimum algorithm compared with cytotoxigenic culture was glutamate dehydrogenase enzyme immunoassay–nucleic acid amplification test, with 94·6% sensitivity, but specificity was less than 99% (appendix). The combination of toxin enzyme immunoassay 2 and nucleic acid amplification test was best for reproduction of the cell cytotoxin assay result, with a high specificity but comparatively low sensitivity (appendix). Overall, the AUROC was higher for two-stage algorithms than for individual assays. The performance of glutamate dehydrogenase enzyme immunoassay and enzyme immunoassay 2 was almost identical to that of toxin enzyme immunoassay combined with nucleic acid amplification test (appendix) compared with cell cytotoxin assay. We analysed three main algorithms in the testing phase that were optimised for cytotoxigenic culture (glutamate dehydrogenase–nucleic acid amplification test), cell cytotoxin assay (toxin enzyme immunoassay–nucleic acid amplification test), or a compromise (glutamate dehydrogenase enzyme immunoassay–enzyme immunoassay 2).

The poor performance of the individual assays was confirmed when the training and testing datasets were combined (table 5). As in the training set, the optimum algorithm to reproduce cytotoxigenic culture was glutamate dehydrogenase enzyme immunoassay–nucleic acid amplification test, but this algorithm had specificity less than 98·0%. Toxin enzyme immunoassay 2–nucleic acid amplification test and glutamate dehydrogenase enzyme immunoassay–toxin enzyme immunoassay 2 were almost identical in performance to cell cytotoxin assay (table 5).

**Table 5:** Sensitivity and specificity of individual assays and algorithms compared with both reference methods

	**Cytotoxigenic culture**						**Cytotoxin assay**					
	GDH EIA	Toxin*EIA 1	Toxin EIA 2	GDH EIA NAAT	Toxin EIA 2 NAAT	GDH toxin EIA 2	GDH EIA	Toxin† EIA 1	Toxin EIA 2	GDH EIA NAAT	Toxin EIA 2 NAAT	GDH toxin EIA 2
Sensitivity (%; 95% CI)	94·5% (92·9–95·8)	45·6% (42·0–49·1)	58·0% (55·0–61·1)	91·5% (89·6–93·1)	57·8% (54·8–60·9)	57·0% (53·9–60·0)	96·4% (94·8–97·7)	66·9% (62·7–70·8)	83·2% (80·3–85·8)	95·6% (93·9–97·0)	82·9% (80·0–85·6)	81·8% (78·8–84·5)
Specificity (%; 95% CI)	94·5% (94·1–94·9)	99·2% (99·0–99·4)	98·7% (98·4–98·9)	98·0% (97·7–98·3)	99·5% (99·3–99·6)	99·4% (99·3–99·6)	92·2% (91·7–92·7)	99·3% (99·1–99·5)	98·8% (98·6–99·0)	95·9% (95·6–96·3)	99·6% (99·4–99·7)	99·5% (99·4–99·6)
PPV (%; 95% CI)	61·0% (58·6–63·4)	84·5% (80·7–87·8)	80·0% (77·0–82·8)	80·7% (78·3–82·9)	90·7% (88·3–92·8)	90·1% (87·5–92·2)	43·9% (41·4–46·3)	86·4% (82·8–89·6)	81·2% (78·2–83·9)	59·7% (56·8–62·5)	92·1% (89·8–94·0)	91·6% (89·2–93·6)
NPV (%; 95% CI)	99·5% (99·3–99·6)	95·2% (94·7–95·6)	96·3% (95·9–96·6)	99·2% (99·0–99·4)	96·3% (95·9–96·6)	96·2% (95·8–96·5)	99·8% (99·6–99·8)	97·9% (97·6–98·2)	98·9% (98·7–99·1)	99·7% (99·6–99·8)	98·9% (98·7–99·1)	98·9% (98·7–99·0)

n=12 420, although small variations in n for each test or algorithm are shown in the appendix. GDH=glutamate dehydrogenase. EIA=enzyme immunoassay. NAAT=nucleic acid amplification test. PPV=positive predictive value. NPV=negative predictive value.

* n=9191 because some centres continued to use the assay in the testing phase. Per-protocol version is in the appendix.

† n=9160 because some centres continued to use the assay in the testing phase. Per-protocol version is in the appendix.

## Discussion

We clinically validated the laboratory diagnosis of *C difficile* infection according to established but divergent reference methods in the largest diagnostic study of this disease so far (panel). Diarrhoea is a common symptom but is rarely due to *C difficile.*^7^ We noted that more than 90% of diarrhoeal samples submitted had no evidence of *C difficile* infection. However, the 30-day all-cause mortality rate was higher than 8% in patients with diarrhoea, indicating a group with severe underlying disease. The presence of cytotoxigenic *C difficile* in faeces of asymptomatic patients varies from roughly 2% in the general population^28^, ^29^ to up to 7–25% of patients admitted to hospital.^30^, ^31^ Thus, patients carrying *C difficile* only as a “bystander” (ie, asymptomatically colonised patients) who develop health-care-associated diarrhoea for other reasons would have a comparatively high case-fatality rate, which would necessitate a large validation study.

Panel **Research in context**

**Background**

The clinical interpretation of tests for *C difficile* infection is confused by uncertainty about which of the two reference methods correlates best with clinical outcome. We have done the largest study of its type to validate the most clinically appropriate reference method for *C difficile* infection. This issue has previously been addressed only in small studies.^25^, ^26^ Typically, studies of *C difficile* diagnostic tests have not included clinical outcome measures, and thus positive results are assumed to correlate with true disease.^9^, ^12^, ^15^

**Interpretation**

We have shown that in more than 6000 patients with diarrhoea, no increase in mortality occurred when a toxigenic *C difficile* strain alone was present (cytotoxigenic culture positive, cell cytotoxin assay negative). By contrast, toxin (cell cytotoxin assay) positivity was associated with clinical outcome, and so this reference method best defines true cases of *C difficile* infection. Other clinical indicators were worse for cell cytotoxin assay-positive cases, but noted no difference between cytotoxigenic culture-positive, cell cytotoxin assay-negative cases, and negative controls.

For the first time, we have described a new diagnostic category of potential *C difficile* excretors (cytotoxigenic culture positive and cell cytotoxin assay negative) to characterise patients with diarrhoea that is unlikely to be due to *C difficile* infection, but who nevertheless can cause cross-infection. No single assay adequately reproduced either reference method as a standalone test. Consequently, we identified *C difficile* infection test algorithms that are optimised according to either cytotoxigenic culture results (glutamate dehydrogenase–nucleic acid amplification test) or cell cytotoxin assay results (enzyme immunoassay 2–nucleic acid amplification test). A compromise solution to *C difficile* infection testing is to use glutamate dehydrogenase (or nucleic acid amplification test–toxin enzyme immunoassay 2). In so doing, a highly sensitive first-stage *C difficile* test (glutamate dehydrogenase or nucleic acid amplification test) allows detection of patients colonised by *C difficile* (but without free toxin), whereas the second more specific test, which needs to be done only on a few faecal samples, identifies those likely to have *C difficile* infection. Importantly, in agreement with a recent single-centre study,^27^ we reported that use of a nucleic acid amplification test alone leads to over-diagnosis of *C difficile* infection, as evidenced by an absence of association with mortality and *C difficile* infection-related complications.

We noted a higher case-fatality rate in cases that were cell cytotoxin assay positive (group 1) than in those that were cytotoxigenic culture positive and cytotoxin assay negative (group 2) and those that were negative by both reference methods (group 3). Patients with a positive cytotoxigenic culture but negative cell cytotoxin assay had the same case-fatality rate as did *C difficile*-negative cases; indeed, their fatality rate per 1000 bed days was not significantly lower than that noted for *C difficile*-negative patients. These results indicate that *C difficile* infection is confirmed by a positive cell cytotoxin assay and not by cytotoxigenic culture, which supports the findings of earlier smaller studies^32^, ^33^ but contrasts with others.^34^ Differences in case-fatality rates between sites (Table 1, Table 2, Table 3, Table 4) are probably attributable to differences in case mixes and endorse the need for a large multicentre study. Although most deaths were in patients with negative tests for *C difficile*, and so were unrelated to *C difficile* infection, the presence of toxin in faecal samples was still associated with a fatal outcome in multivariate analysis. Since many patients without *C difficile* have several comorbidities, we could not differentiate between patients on the basis of our predefined markers of severity.

The two reference methods provide different information. A positive cell cytotoxin assay indicates that the diarrhoea was probably caused by *C difficile* infection, whereas a positive cytotoxigenic culture indicates that a patient could be infectious even though the diarrhoea might have resulted from another cause. Since we recorded no evidence of ongoing carriage, we propose the term “potential *C difficile* excretor” for patients with samples that are cytotoxigenic culture positive but cell cytotoxin assay negative. By analysis of cases with discordant reference method results, we showed that poor outcome correlated with detectable toxin as opposed to the presence of *C difficile* with toxigenic potential, whether or not specific *C difficile* infection treatment was given. Since the median duration of diarrhoea was 2 days in these patients, most symptoms probably resolved before cytotoxigenic culture results were available, with treatment considered unnecessary. Our findings indicate the need to treat patients who are positive for *C difficile* toxin. The management of potential *C difficile* excretors is less clear, but, because they might be infectious, infection control precautions should be taken. When *C difficile* infection is excluded, other causes of diarrhoea should be sought. We caution, however, that the need for *C difficile* infection treatment is a clinical decision, which might be improved with clinical interpretation of a result,^35^ in view of the suboptimum sensitivity of existing assays.^14^, ^15^ Since only 25% of cases of *C difficile* infection could be matched with previous cases,^36^ the sources of *C difficile* in some of the remaining unexplained cases could be patients who are colonised by toxigenic strains. These patients could be asymptomatic or, as we identified, patients with diarrhoea not due to *C difficile* infection (ie, potential *C difficile* excretors). However, since these tests are not being used as screening tests, we cannot be sure how much of an effect the identification of *C difficile*-colonised patients would have on *C difficile* transmission rates in hospitals.

In the UK, the long-standing policy is to take samples from all patients with diarrhoea, even after only one or two episodes, and more tests for *C difficile* infection are done than in other European countries.^37^ Outcome data were nearly complete, and although the data available for secondary endpoints such as treatment were available in 60–70% of cases, the results seem robust. As an observational study, the strong associations shown here do not confirm causality. However, the fact that patients who were cytotoxigenic culture positive but cell cytotoxin assay negative had good outcomes, despite not receiving any specific treatment for *C difficile* infection, strongly suggests a causal link between outcome and cell cytotoxin assay positivity.

For convenience, most diagnostic laboratories do not use reference methods. However, the performance of routine *C difficile* diagnostic tests is established by comparing them to reference methods. Our data emphasise the need to choose the appropriate reference method for each test. This multicentre study draws attention to geographical variation in test performance (appendix) and confirms previous study findings of poor positive predictive values for toxin enzyme immunoassays.^14^, ^15^

The sensitivities of the standalone glutamate dehydrogenase enzyme immunoassay and Xpert nucleic acid amplification test were not as high as have been previously reported.^17^, ^18^, ^19^, ^20^ However, the large sample size in this study has produced a more accurate result with narrower confidence intervals. The negative predictive values of these two assays were greater than 99% compared with either reference method, but positive predictive values were less than 75%, at best (appendix). Although the Xpert nucleic acid amplification test detects only toxigenic *C difficile*, unlike the glutamate dehydrogenase assay, the poor positive predictive values that we noted indicate that this assay should not be used alone to diagnose *C difficile* infection. Therefore, our data contradict a recent recommendation by Surawicz and colleagues^38^ to use a nucleic acid amplification test alone. Indeed, cases of *C difficile* infection diagnosed by this test alone have been shown to be significantly less likely to be associated with complications than those diagnosed by a combination of nucleic acid amplification test and toxin assay.^27^ Notably, laboratory diagnosis by nucleic acid amplification test as opposed to detection of toxin (cell cytotoxin assay), according to our data would yield 81% (95% CI 77–85) more positive results. The longer turnaround times and high cost of two-stage algorithms have led some researchers to advocate nucleic acid amplification test alone despite the potential disadvantages.^11^, ^12^, ^38^ Our results show that two-stage algorithms can improve the accuracy of diagnosis of *C difficile* infection (figure 2). The positive predictive values and negative predictive values of the algorithms will vary dependent on the prevalence of *C difficile* infection in the samples tested. However, standalone tests would be unlikely to have acceptable performance unless the prevalence is very high (>40%; appendix).

Since the two reference methods are clearly not comparable, no single algorithm could be optimised for both. Glutamate dehydrogenase enzyme immunoassay–nucleic acid amplification test and toxin enzyme immunoassay 2–nucleic acid amplification test best reproduced cytotoxigenic culture and cell cytotoxin assay, respectively. Since the clinical implications of a positive cytotoxigenic culture and cell cytotoxin assay differ, to undertake both of these algorithms is complex and expensive. However, another algorithm (glutamate dehydrogenase enzyme immunoassay–toxin enzyme immunoassay 2) performed almost identically to toxin enzyme immunoassay 2–nucleic acid amplification test in prediction of cell cytotoxin assay. This algorithm has the advantage that it allows the stratification of results into three categories: *C difficile* infection positive, potential *C difficile* excretor, and *C difficile* infection negative. This advantage allows rapid reporting of about 86% of results as glutamate dehydrogenase negative, which has important implications for infection control; the high negative predictive value (99·5% for cytotoxigenic culture and and 99·8% for cell cytotoxin assay; table 5) associated with this test means that *C difficile* infection can be excluded with a high degree of certainty. Then reflex testing for toxin enzyme immunoassay allows the identification of samples that are cell cytotoxin assay positive, with a sensitivity of just above 80%. The remaining samples with discordant results (glutamate dehydrogenase enzyme immunoassay positive/toxin enzyme immunoassay negative)—about 8% of samples in this study—can then be managed. In our sample, about 60% of these cases were negative, 30% potential *C difficile* excretors, and 10% positive for cell cytotoxin assay. These discordant cases can be treated as negative or potential *C difficile* excretors, despite the relatively low positive predictive value. The final possibility is to do a nucleic acid amplification test on these discordant samples to clearly identify potential *C difficile* excretors. The three-stage strategy effectively combines the detection of cell cytotoxin assay and cytotoxigenic culture into one algorithm.

Notwithstanding the size of this study and completeness of the follow-up, the reproduction of real-life testing in diagnostic laboratories in England has potential limitations, in addition to its strengths. This trial was an observational study in which the laboratory workers were not masked to results. However, in view of the time taken to obtain results for the reference methods and the length of follow-up, the results were unlike to have affected, or to have been affected by, the results of tests done on day 1. The other serendipitous result of waiting for several days for results of culture was that many patients who were positive only by cytotoxigenic culture were not treated for *C difficile* infection, which helps to confirm the safety of this strategy when undertaken with clinical supervision. The frequency of stool sampling and testing was high. The application of these findings to health-care settings in which testing is less frequent and needs to be requested by physicians is uncertain. The strategy of testing only physician-requested samples certainly misses many cases of *C difficile* infection.^39^ To imagine how a different selection of cases to sample or test for *C difficile* infection would affect the clinical meaning of the reference methods is difficult.

We have shown that detection of toxin is an essential step in the diagnosis of *C difficile* infection. The importance of toxin detection, and also the deficiencies of existing tests, should drive further assay development. We have identified the best possible algorithm for *C difficile* infection diagnosis that also identifies potential *C difficile* excretors. Further investigation is needed to establish the clinical implications of such patients, including their infection risk to others and their optimum management.
