# Supplementary material for: Differences in outcome according to Clostridium difficile testing method: a prospective multicentre diagnostic validation study of C difficile infection
Source: Lancet Infect Dis. 2013 Nov;13(11):936–45. doi: 10.1016/S1473-3099(13)70200-7 (PMC3822406; doi:10.1016/S1473-3099(13)70200-7)
Supplement: Supplementary appendix [file mmc1.pdf]

THE LANCET Infectious Diseases

Supplementary webappendix

This webappendix formed part of the original submission and has been peer reviewed. We post it as supplied by the authors.

Supplement to: Planche TD, Davies KA, Coen PG, et al. Differences in outcome according to Clostridium difficile testing method: a prospective multicentre diagnostic validation study of *C difficile* infection. *Lancet Infect Dis* 2013; published online Sep 3. http://dx.doi.org/10.1016/S1473-3099(13)70200-7.

Additional Materials

Additional Laboratory Methods

Cell Cytotoxin assay (CTA)

Faecal samples were diluted 1:5 in phosphate buffered saline and centrifuged at 16000g before 20µl of supernatant were added to duplicate Vero cell monolayers, one set of which was protected by addition of 20 µl *C. sordelli* antitoxin (Prolab Diagnostics, UK). Vero cells (European collection of animal cell cultures) were grown in 96-well flat bottomed microtitre trays (VWR, UK) in 160 µl of Dulbeco medium (Invitrogen, UK) Samples were filtered before testing if supernatant was cloudy. A positive result was recorded if cell rounding was seen in >50% of the unprotected cells only, after 24 or 48 hours of incubation at 37°C in a CO2 incubator[1].

Cytotoxigenic culture (CC)

Samples were cultured, following alcohol shock in 50:50 v/v absolute ethanol and water, on Braziers' agar (Oxoid, UK). Plates were incubated in an anaerobic workstation (Leeds, London) or anaerobic jars (Oxford) and inspected for growth after 48 h. Suspect colonies were confirmed by presence of green/yellow fluorescence under UV light (365nm) and *C. difficile* latex agglutination (Microgen Bioproducts Ltd, Camberley, UK). Isolates were inoculated into brain heart infusion broth (Oxoid, Basingstoke, UK) and incubated anaerobically for 48 h. Culture supernatants were tested by CTA as above[2].

Laboratory Methodologies

The EIAs were automated and performed on DS2 instruments (Magellan Biosciences, USA); optical densities were read on dual wavelengths at 450/620nm. The following assays were evaluated: Techlab *C. difficile* Tox A/B II toxin EIA (toxin EIA 2) (Techlab, USA) with a cut-off of 0·08; Meridian Premier toxins A&B EIA (toxin EIA 1) (Meridian, USA) with a cut off of 0·1; and the Techlab C. diff Chek-60 (TGDH) GDH EIA (Techlab, USA) with a cut-off of 0·08. There is no equivocal zone for any of these three EIAs. The NAAT assay GeneXpert *C. difficile* (Xpert) was performed using the GeneXpert (Cepheid, USA). Samples with invalid results were repeated once and if still invalid were recorded as such. Assays and reference methods were performed on the same day by the same evaluator at three sites (Leeds, London) or two evaluators in one site (Oxford). In total, one evaluator worked at each of the two London sites, two at Leeds, and three at Oxford

PCR-ribotyping

PCR-ribotyping was performed on all isolates following the *Clostridium difficile* Ribotyping Network of England and Northern Ireland protocol[3].

Statistical Methods

For sample size calculations for the laboratory assessment we assumed testing algorithm sensitivity and specificity of 90% and 99·5%, respectively, with 4·5% of samples positive; thus, 8,000-10,000 specimens will estimate sensitivity within 3% and specificity within 0·2%.

The rationale is that for a randomly sampled (negative, positive) reference test pair, the AUROC is the probability that the test (t) ranks a true positive as more likely infected than a true negative (i.e. $t_p > t_n$). For each test algorithm we used 1000 bootstrap samples for the AUROC, estimated via randomly selected record pairs, (t_p, t_n); the proportion of pairs where $t_p > t_n$ is the AUROC. For testing the significance of difference between two AUROCs we used the distributional form of their difference coming from 2000 bootstrap samples. The Boostrap sample size of 1000 was chosen for consistency in the estimates with a standard error within 0·1% of the estimate

Additional results

The toxin EIA 1 and Xpert assays were not used first line in the testing phase, and so represent a smaller and partially selected dataset (table 1); the toxin EIA 1 assay was used during the testing phase at one site (n=2558) as this was the routine test there. In the training phase (n=6753) 389, 559 and 704 samples were CTA, CC and NAAT positive.

The sensitivity of toxin EIA 1 and toxin EIA 2 was 66·9% (62·7-70·8% 95% CI) and 83·2% (80·3-85·8% 95% CI), respectively, compared with CTA. Toxin EIA 1 also showed the highest variability between sites when AUROCs were examined, further demonstrating the poor performance of this test (table 4).

Episodes with missing clinical or death data are more likely to be female, older and have been in hospital for longer (p<0.0001): median age 74 (missing) vs 68 years of age (with data); 62% female (missing) vs 53% (with data), median los (at testing) 6 days (missing) 5 days (with data).

As some patients were tested more than once, we needed to check for within-patient correlation in the results. We did this by means of multi-level analysis through a logistic regression model where the outcome was either one of the gold standard tests (cytotoxigenic culture or cytotoxin test) and found that the random effects model invariably fitted the data significantly better than the random effects model, with large intraclass correlation coefficients (ICC). We compared negative twice the difference in log-likelihoods between models against the Chi-square distribution to assess the fit to the data, as advocated by Twisk[4]. When we used the deduplicated data set, where samples for the same episode were removed, the ICC became non-significant. Repeating the analyses using just the deduplicated set of episodes (within a 28 day window), did not lead to important changes in the results, although the standard errors were slightly larger". A "real world" clinical laboratory will be using multiple samples for each patient and this was an additional reason for keeping the main table in the manuscript showing the results for the full samples as received by the laboratory.

Ribotyping

The isolates comprised 114 different PCR-ribotypes. Ten PCR-ribotypes accounted for 63% of all of the isolates, which were (in rank order) 015, 014, 027, 002, 010, 005, 078, 140, 026, and 020.

Inter-laboratory variation

The monthly quality assurance samples yielded no discordant results between laboratories. There were variations in performance of each assay across the laboratories during the training phase shown by AUROC analysis (table 4). The largest inter-site variation was seen with the toxin EIA 1 (coefficient of variation = 8·08% and 7·35% for CTA and CC, respectively). The correlation between the positivity rates of each assay over the time of the study was fairly consistent (figure 4). Time series plots showing the positivity rate of each assay during the study by site showed the same stacking pattern of the assays and confirmed that intra- and inter-site variability mirrored that seen in the study as a whole (data not shown).

Previous studies have often been single centre and so have been unable to determine inter-laboratory variation in performance between commercial assays and have been subject to variable strain distributions, which may introduce bias. Inter-site variation in this study was demonstrated by AUROC analysis (table 4). Indeed, if performed only at St George's (n= 1593) for example, toxin EIA 1 had the highest AUROC of all single assays in the testing phase compared with CTA, in contrast with overall results. The reasons for this variability are manifold, and could include the prevalence of PCR-

ribotypes [5]. However, only ten PCR-ribotypes represented 63% of all study isolates, and PCR-ribotypes 014 and or 015 were found amongst the three most common types at each site. Comparing positivity rates for each assay at each centre showed the same trends in variability between sites. This indicates that intrinsic assay factors are likely to be affecting performance.

Table 1. Results of individual assays compared with both reference methods

Reference assay	Assay	Total Number	True positive	True negative	False positive	False negative	Invalid	Test not performed	Reasons for not testing
Cell-cytotoxin n = 12366	GDH EIA	12329	705	10696	902	26	0	37	**Failure of DS2 at one site/not enough sample**
	Toxin EIA 1	9160[a]	363	8560	58	180	0	3206	**Excluded from testing phase due to poor performance in training phase/ not enough sample**
	Toxin EIA 2	12333	608	11461	141	123	0	33	**Failure of DS2 at one site/not enough sample/ not enough sample**
	Toxin gene PCR	8792	713	7502	561	16	26	3548	**Only included in testing set as second line assay/ not enough sample**
Cytotoxigenic culture n = 12402	GDH EIA	12365	977	10707	624	57	0	37	**Failure of DS2 at one site/not enough sample/ not enough sample**
	Toxin EIA 1	9191[b]	355	8347	66	424	0	3211	**Excluded from testing phase due to poor performance in training phase/ not enough sample**
	Toxin EIA 2	12369	600	11185	150	434	0	33	**Failure of DS2 at one site/not enough sample/ not enough sample**
	Toxin gene PCR	8827	971	7497	303	56	26	3549	**Only included in testing set as second line assay/ not enough sample**

[a] One site continued to use assay in testing phase n =2558

[b] One site continued to use assay in testing phase n =2559

Table 2A. Sensitivity and Specificity of individual assays and algorithms compared with both reference methods – Training dataset (n = 6753)

	**Cell-cytotoxicity**								
	**Single assays-Manufacturers' cut-offs**				**Two stage assays-Manufacturers' cut-offs**				
	GDH EIA	**NAAT**	**Toxin EIA 1**	**Toxin EIA 2**	**GDH EIA**	**GDH EIA**	**GDH EIA**	**Toxin EIA 1**	**Toxin EIA 2**
					**Toxin EIA 1**	**NAAT**	**Toxin EIA 2**	**NAAT**	**NAAT**
Sensitivity % (95% CI)	95.9 (93.4-97.6)	96.9 (94.7-98.4)	69.2 (64.3-73.8)	82.3 (78.1-85.9)	67.4 (62.4-72.1)	94.6 (91.9-96.6)	80.5 (76.2-84.3)	69.0 (64.0-73.6)	82.0 (77.8-85.7)
Specificity % (95% CI)	92.1 (91.4-92.8)	94.9 (94.3-95.4)	99.4 (99.2-99.6)	98.8 (98.4-99.0)	99.7 (99.5-99.8)	96.0 (95.5-96.5)	99.6 (99.4-99.7)	99.7 (99.6-99.8)	99.6 (99.4-99.8)
PPV% (95% CI)	42.8 (39.5-46.2)	54.0 (50.2-57.8)	87.4 (83.1-90.9)	81.4 (77.2-85.1)	93.1 (89.4-95.8)	59.3 (55.3-63.2)	91.8 (88.4-94.5)	93.9 (90.4-96.4)	93.0 (89.8-95.5)
NPV% (95% CI)	99.7 (99.6-99.8)	99.8 (99.6-99.9)	98.1 (97.8-98.5)	98.9 (98.6-99.1)	98.0 (97.7-98.4)	99.7 (99.5-99.8)	98.8 (98.5-99.1)	98.1 (97.8-98.4)	98.9 (98.6-99.1)
	**Cytotoxigenic culture**								
	**Single assays-Manufacturers' cut-offs**				**Two stage assays-Manufacturers' cut-offs**				
	**GDH EIA**	**NAAT**	**Toxin EIA 1**	**Toxin EIA 2**	**GDH EIA**	**GDH EIA**	**GDH EIA**	**Toxin EIA 1**	**Toxin EIA 2**
					**Toxin EIA 1**	**NAAT**	**Toxin EIA 2**	**NAAT**	**NAAT**
Sensitivity % (95% CI)	93.6 (91.2-95.4)	93.2 (90.8-95.1)	47.0 (42.7-51.3)	56.7 (52.5-60.9)	45.9 (41.6-50.2)	90.0 (87.2-92.3)	55.5 (51.2-59.6)	46.8 (42.5-51.1)	56.5 (52.3-60.7)
Specificity % (95% CI)	94.5 (93.9-95.0)	96.9 (96.5-97.4)	99.2 (99.0-99.4)	98.8 (98.4-99.0)	99.6 (99.4-99.7)	98.1 (97.7-98.4)	99.5 (99.3-99.6)	99.6 (99.4-99.7)	99.5 (99.3-99.7)
PPV % (95% CI)	60.5 (57.2-63.8)	74.7 (71.4-77.9)	84.7 (80.1-88.6)	80.4 (76.2-84.3)	90.5 (86.5-93.7)	81.0 (77.7-84.0)	90.6 (87.0-93.5)	91.0 (87.1-94.1)	91.9 (88.5-94.5)
NPV % (95% CI)	99.4 (99.2-99.6)	99.4 (99.1-99.6)	95.4 (94.9-96.0)	96.2 (95.7-96.6)	95.4 (94.8-95.9)	99.1 (98.8-99.3)	96.1 (95.6-96.6)	95.4 (94.9-96.0)	96.2 (95.7-96.7)

Table 2B. Sensitivity and Specificity of individual assays and algorithms compared with both reference methods – Testing dataset (n = 5667)

	**Cell-cytotoxicity**								
	**Single assays-Manufacturers' cut-offs**				**Two stage assays-Manufacturers' cut-offs**				
	GDH EIA	**NAAT**	**Toxin EIA 1**	**Toxin EIA 2**	**GDH EIA**	**GDH EIA**	**GDH EIA**	**Toxin EIA 1**	**Toxin EIA 2**
					**Toxin EIA 1**	**NAAT**	**Toxin EIA 2**	**NAAT**	**NAAT**
Sensitivity % (95% CI)	97.1 (94.5-98.5)	98.8 (96.8-99.6)	61.3 (53.4-68.7)	84.2 (79.8-87.8)	59.4 (51.5-66.9)	96.8 (94.1-98.3)	83.3 (78.9-87.0)	61.3 (53.4-68.8)	83.9 (79.5-87.6)
Specificity % (95% CI)	92.4 (91.6-93.1)	86.5 (84.8-88.0)	99.2 (98.7-99.5)	98.7 (98.4-99.0)	99.9 (99.7-99.9)	95.9 (95.3-96.4)	99.5 (99.2-99.7)	99.7 (99.4-99.9)	99.5 (99.2-99.6)
PPV% (95% CI)	45.1 (41.5-48.8)	58.3 (54.2-62.4)	84.0 (75.9-89.9)	80.9 (76.3-84.8)	93.3 (86.3-97.1)	60.2 (55.9-64.3)	91.3 (87.5-94.1)	93.5 (86.5-97.1)	91.1 (87.3-93.9)
NPV% (95% CI)	99.8 (99.6-99.9)	99.7 (99.3-99.9)	97.4 (96.7-98.0)	99.0 (98.7-99.2)	98.7 (98.3-99.0)	99.8 (99.6-99.9)	98.9 (98.6-99.2)	97.4 (96.7-98.0)	99.0 (98.6-99.2)
	**Cytotoxigenic culture**								
	**Single assays-Manufacturers' cut-offs**				**Two stage assays-Manufacturers' cut-offs**				
	**GDH EIA**	**NAAT**	**Toxin EIA 1**	**Toxin EIA 2**	**GDH EIA**	**GDH EIA**	**GDH EIA**	**Toxin EIA 1**	**Toxin EIA 2**
					**Toxin EIA 1**	**NAAT**	**Toxin EIA 2**	**NAAT**	**NAAT**
Sensitivity % (95% CI)	95.6 (93.2-97.2)	96.2 (93.9-97.6)	42.4 (36.0-49.0)	59.6 (55.0-64.0)	39.5 (33.4-45.0)	93.3 (90.5-95.3)	58.7 (54.2-63.2)	41.9 (35.5-48.5)	58.6 (54.0-63.1)
Specificity % (95% CI)	94.5 (93.8-95.1)	92.3 (90.1-93.5)	99.2 (98.7-99.5)	98.6 (98.2-98.9)	99.8 (99.6-99.9)	97.9 (97.5-98.3)	99.4 (99.1-99.6)	99.6 (99.2-99.8)	99.4 (99.2-99.6)
PPV % (95% CI)	61.6 (58.0-65.1)	78.0 (74.3-81.2)	84.0 (75.9-89.9)	79.5 (74.8-83.5)	91.4 (83.9-95.8)	80.4 (76.8-93.6)	89.4 (85.3-92.5)	91.6 (84.2-95.8)	90.1 (86.0-93.1)
NPV % (95% CI)	99.6 (99.3-99.7)	98.8 (98.1-99.3)	94.4 (93.4-95.3)	96.4 (95.8-96.8)	97.1 (96.6-97.6)	99.4 (99.1-99.6)	96.3 (95.8-96.8)	94.4 (93.4-95.3)	96.4 (95.8-96.8)

Table 3A. Sensitivity and Specificity of individual assays and algorithms compared with both reference methods – Training set, first sample of episodes only (n = 5,735, although small variations in n for each test/algorithm shown in table 1)

	**Cytotoxigenic culture**						**Cytotoxin**					
	**GDH EIA**	**Toxin[a] EIA 1**	**Toxin EIA 2**	**GDH EIA**	**Toxin EIA 2**	**GDH**	**GDH EIA**	**Toxin[b] EIA 1**	**Toxin EIA 2**	**GDH EIA**	**Toxin EIA 2**	**GDH**
				**NAAT**	**NAAT**	**Toxin EIA 2**				**NAAT**	**NAAT**	**Toxin EIA 2**
Sensitivity %	94.1	48.4	58.8	90.5	58.6	57.7	96.2	68.2	81.3	94.8	81.0	79.9
(95% CI)	(91.6-96.0)	(43.7-53.0)	(54.2-63.2)	(87.5-93.0)	(54.0-63.0)	(53.1-62.2)	(93.6-98.0)	(63.0-73.2)	(76.8-85.3)	(91.8-96.9)	(76.5-85.1)	(75.2-84.0)
Specificity %	94.5	99.3	98.8	98.2	99.5	99.5	92.4	98.9	98.8	96.3	99.6	99.5
(95% CI)	(93.9-95.1)	(99.0-99.5)	(98.5-99.1)	(97.8-98.5)	(99.3-99.7)	(99.3-99.7)	(91.7-93.1)	(98.5-99.1)	(98.6-99.0)	(95.8-96.8)	(99.4-99.7)	(99.3-99.7)
PPV %	60.6	85.8	81.3	81.4	92.0	91.0	43.9	81.8	81.2	61.9	92.7	91.6
(95% CI)	(57.0-64.2)	(81.0-89.8)	(76.7-85.3)	(77.8-84.6)	(88.4-94.8)	(87.2-94.0)	(44.7-48.3)	(77.3-85.8)	(78.2-83.9)	(57.6-66.1)	(89.1-95.3)	(87.9-95.5)
NPV %	99.4	95.6	96.4	99.1	96.4	96.3	99.7	98.8	98.9	99.7	98.8	98.7
(95% CI)	(99.2-99.6)	(95.0-96.1)	(95.9-96.9)	(98.9-99.4)	(95.9-96.9)	(95.8-96.8)	(99.6-99.9)	(98.5-99.1)	(98.7-99.1)	(99.5-99.8)	(98.5-99.1)	(98.4-99.0)

[a]n= 5697 as some centres continued using the assay in the testing phase. Per protocol version shown in table 2.

[b]n = 5672 as some centres continued using the assay in the testing phase. Per protocol version shown in table 2.

Table 3B. Sensitivity and Specificity of individual assays and algorithms compared with both reference methods – Testing set, first sample of episodes only (n = 4,899, although small variations in n for each test/algorithm shown in table 1)

	**Cytotoxigenic culture**						**Cytotoxin**					
	**GDH EIA**	**Toxin[a] EIA 1**	**Toxin EIA 2**	**GDH EIA**	**Toxin EIA 2**	**GDH**	**GDH EIA**	**Toxin[b] EIA 1**	**Toxin EIA 2**	**GDH EIA**	**Toxin EIA 2**	**GDH**
				**NAAT**	**NAAT**	**Toxin EIA 2**				**NAAT**	**NAAT**	**Toxin EIA 2**
Sensitivity %	95.7	38.8	59.1	93.2	58.8	58.1	97.8	61.5	85.1	97.5	84.8	84.1
(95% CI)	(93.2-97.5)	(31.7-46.3)	(54.1-64.0)	(90.2-95.5)	(53.8-63.7)	(53.0-63.0)	(95.3-99.2)	(52.2-70.1)	(80.4-89.1)	(94.8-99.0)	(80.0-88.8)	(79.2-88.2)
Specificity %	94.6	99.2	98.6	98.0	99.4	99.4	92.4	99.5	98.7	95.9	99.5	99.5
(95% CI)	(93.9-95.2)	(98.7-99.6)	(98.3-99.0)	(97.5-98.4)	(99.1-99.6)	(99.1-99.6)	(91.6-93.1)	(99.0-99.7)	(98.3-99.0)	(95.3-96.4)	(99.2-99.6)	(99.2-99.7)
PPV %	61.0	82.6	79.3	80.4	90.0	89.8	43.5	87.2	79.7	58.7	90.3	90.6
(95% CI)	(57.1-64.9)	(72.9-89.9)	(74.2-83.8)	(76.5-83.9)	(76.5-83.9)	(85.5-93.3)	(39.6-47.6)	(78.3-93.4)	(74.6-84.1)	(54.1-63.3)	(86.1-93.7)	(86.4-93.9)
NPV %	99.6	94.5	96.5	99.4	99.4	96.4	99.9	97.7	91.1	99.8	99.1	99.0
(95% CI)	(99.4-99.8)	(93.4-95.5)	(95.9-97.0)	(99.1-99.6)	(99.1-99.6)	(95.8-96.9)	(99.7-99.9)	(96.9-98.3)	(98.8-99.4)	(99.7-99.9)	(98.8-99.3)	(98.7-99.3)

[a]n= 2127 as some centres continued using the assay in the testing phase. Per protocol version shown in table 2.

[b]n = 2126 as some centres continued using the assay in the testing phase. Per protocol version shown in table 2.

Table 4. AUROC analysis of variation of assay performance at each site for both reference methods during the training phase

		Cell-cytotoxin				Cytotoxigenic culture			
**Assay**	**Site**	**AUROC**	**Standard error**	**% positive**	**Coefficient of variation (%)**	**AUROC**	**Standard error**	**% positive**	**Coefficient of variation (%)**
Toxin EIA 1	All sites	0.9061	0.0035			0.7906	0.0050		
	Leeds	0.9369	0.0045	7.12		0.8288	0.0069	8.00	
	UCH	0.7789	0.0123	3.48	8.03	0.7030	0.0140	5.77	7.35
	SGH	0.8773	0.0119	4.32		0.7878	0.0141	8.27	
	Oxford	0.8780	0.0077	5.83		0.7558	0.0105	9.19	
Toxin EIA 2	All sites	0.9494	0.0026			0.8171	0.0046		
	Leeds	0.9646	0.0035	7.12		0.8344	0.0070	8.77	
	UCH	0.8766	0.0097	3.48	7.43	0.7630	0.0124	5.77	4.20
	SGH	0.8366	0.0131	4.32		0.7747	0.0148	8.27	
	Oxford	0.9615	0.0046	5.83		0.7844	0.0095	9.19	
GDH EIA	All sites	0.9371	0.0030			0.9441	0.0028		
	Leeds	0.9379	0.0045	7.12		0.9260	0.0049	8.77	
	UCH	0.9493	0.0062	3.48	4.63	0.9639	0.0058	5.77	1.75
	SGH	0.8595	0.0124	4.32		0.9429	0.0079	8.27	
	Oxford	0.9125	0.0067	5.83		0.9472	0.0053	9.19	
NAAT	All sites	0.9173	0.0033			0.9034	0.0035		
	Leeds	0.9295	0.0048	7.12		0.8971	0.0055	8.77	
	UCH	0.9387	0.0070	3.48	5.39	0.9392	0.0069	5.77	2.94
	SGH	0.8412	0.0130	4.32		0.9002	0.0102	8.27	
	Oxford	0.8790	0.0077	5.83		0.8809	0.0077	9.19	

Table 5. Effect of changing *C. difficile* prevalence on the positive and negative predictive values when the algorithm GDH/Toxin EIA 2 is compared with each reference method. Discordant samples (GDH +ve/ EIA2 –ve) are considered negative. Performance will vary if discordant samples treat differently. Combined dataset (n = 12420)

	**Cell-cytotoxicity**		**Cytotoxigenic culture**	
**Prevalence**	**PPV**	**NPV**	**PPV**	**NPV**
**2%**	77.0	99.6	66.0	99.1
**4%**	87.2	99.2	79.8	98.2
**6%**	91.3	98.8	85.8	97.3
**8%**	93.4	98.4	89.2	96.4
**10%**	94.8	98.0	91.3	95.4

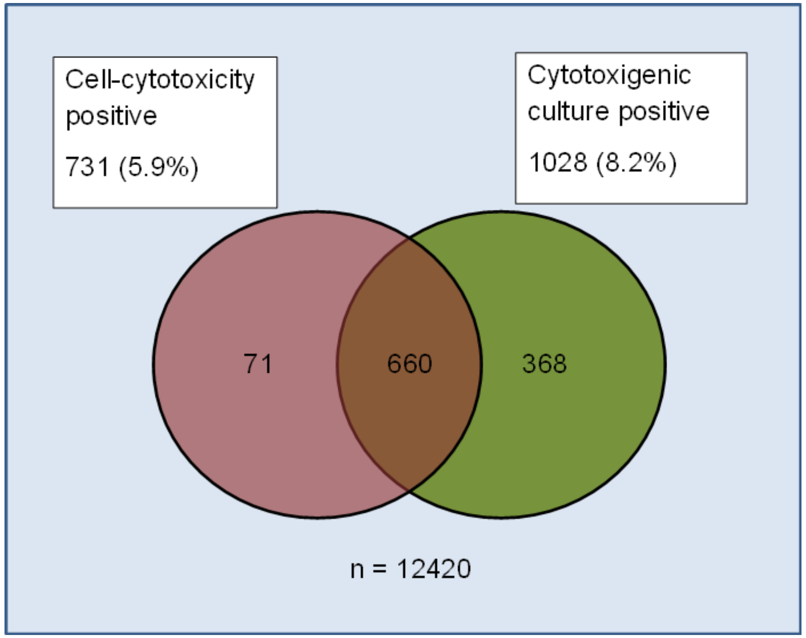

Fig 1. Positive samples by reference method

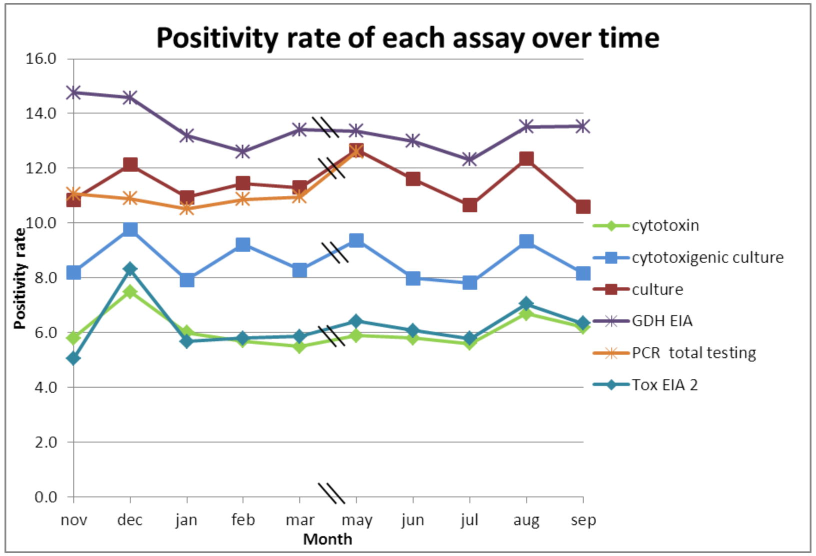

Fig. 4 Time series plot showing the positivity rate of each assay over the study period

References

1. Freeman J, Wilcox MH. The effects of storage conditions on viability of Clostridium difficile vegetative cells and spores and toxin activity in human faeces. *J Clin Pathol* 2003; **56**(2): 126-8.
2. Bouza E, Pelaez T, Alonso R, Catalan P, Munoz P, Creixems MR. "Second-look" cytotoxicity: an evaluation of culture plus cytotoxin assay of Clostridium difficile isolates in the laboratory diagnosis of CDAD. *J Hosp Infect* 2001; **48**(3): 233-7.
3. Stubbs SL, Brazier JS, O'Neill GL, Duerden BI. PCR targeted to the 16S-23S rRNA gene intergenic spacer region of Clostridium difficile and construction of a library consisting of 116 different PCR ribotypes. *J Clin Microbiol* 1999; **37**(2): 461-3.
4. Twisk JWR. Applied Multilevel Analysis A Practical Guide for Medical Researchers. Cambridge: Cambridge University Press; 2006.
5. Tenover FC, Novak-Weekley S, Woods CW, et al. Impact of strain type on detection of toxigenic Clostridium difficile: comparison of molecular diagnostic and enzyme immunoassay approaches. *J Clin Microbiol* 2010; **48**(10): 3719-24.
